# Open-source lab hardware: A versatile microfluidic control and sensor platform

**DOI:** 10.1016/j.ohx.2021.e00229

**Published:** 2021-09-17

**Authors:** Florian Kehl, Vlad F. Cretu, Peter A. Willis

**Affiliations:** a NASA Jet Propulsion Laboratory, California Institute of Technology, Pasadena, CA 91109, USA; b Innovation Cluster Space and Aviation (UZH Space Hub), Air Force Center, University of Zurich, 8600 Dübendorf, Switzerland; c Institute of Anatomy, Faculty of Medicine, University of Zurich, 8057 Zurich, Switzerland; d Institute of Medical Engineering, Space Biology Group, Lucerne University of Applied Sciences and Arts, 6052 Hergiswil, Switzerland

**Keywords:** Microfluidic sample handling, Fluidic sensing, Chemical analysis, Lab automation, Valve controller, Capillary electrophoresis

## Abstract

Here we describe a completely integrated and customizable microfluidic control and sensing architecture that can be readily implemented for laboratory or portable chemical or biological control and sensing applications. The compact platform enables control of 32 solenoid valves, a multitude of pumps and motors, a thermo-electric controller, a pressure controller, and a high voltage power supply. It also features two temperature probe interfaces, one relative humidity and ambient temperature sensor, two pressure sensors, and interfaces to an electrical conductivity sensor, flow sensor, and a bubble detector. The platform can be controlled via an onboard microcontroller and requires no proprietary software.

## Graphical abstract

**Figure:**
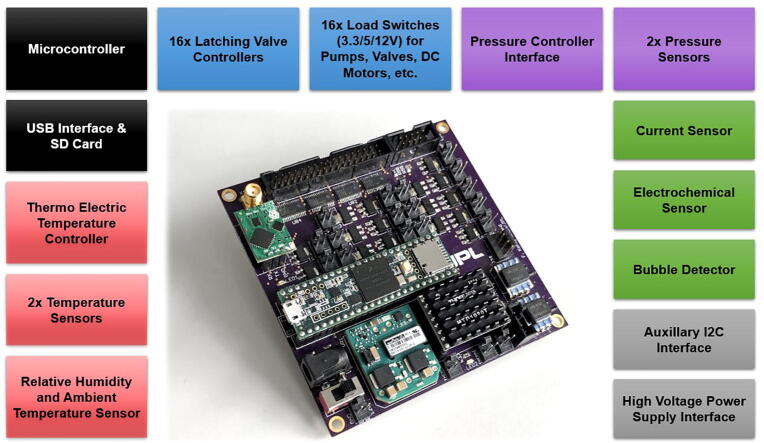

**Table:** 

**Hardware name**	**Microfluidic Control and Sensor Platform (MCSP)**
Subject Area	Chemistry and Biochemistry
Hardware Type	Biological Sample Handling and Preparation
Open Source License	Elsevier User License
Cost of Hardware	Approximate cost of hardware (complete breakdown will be included in the Bill of Materials).$182 USD in base configuration,$383 USD incl. temperature controller, electrochemical and pressure sensors.*
Source File Repository	*https://doi.org/10.17605/OSF.IO/VWHG4*

*The cost information contained in this document is of a budgetary and planning nature and is intended for informational purposes only. It does not constitute a commitment on the part of JPL and/or Caltech.

## Hardware in Context

For the development of nano-, micro-, or mesofluidic experimental setups, lab automation, or lab-on-a-chip applications [1], [2], researchers often have to rely on relatively costly, specialized equipment to control, monitor, and automate their processes. These commercial microfluidic instruments then allow for the control of pumps and valves, and measure parameters such as flow, pressure, and temperature, often only via proprietary software. On the other hand, there are numerous OEM manufacturers of microfluidic components such as valves, pumps, and sensors, but these companies seldom supply the corresponding control electronics required for use. While there has been previous work on open-hardware controllers for microfluidics [3], [4], [5], [6], they were still very application-specific and not generic enough to cover the broad spectrum of potential microfluidic applications.

**Fig. 1:**
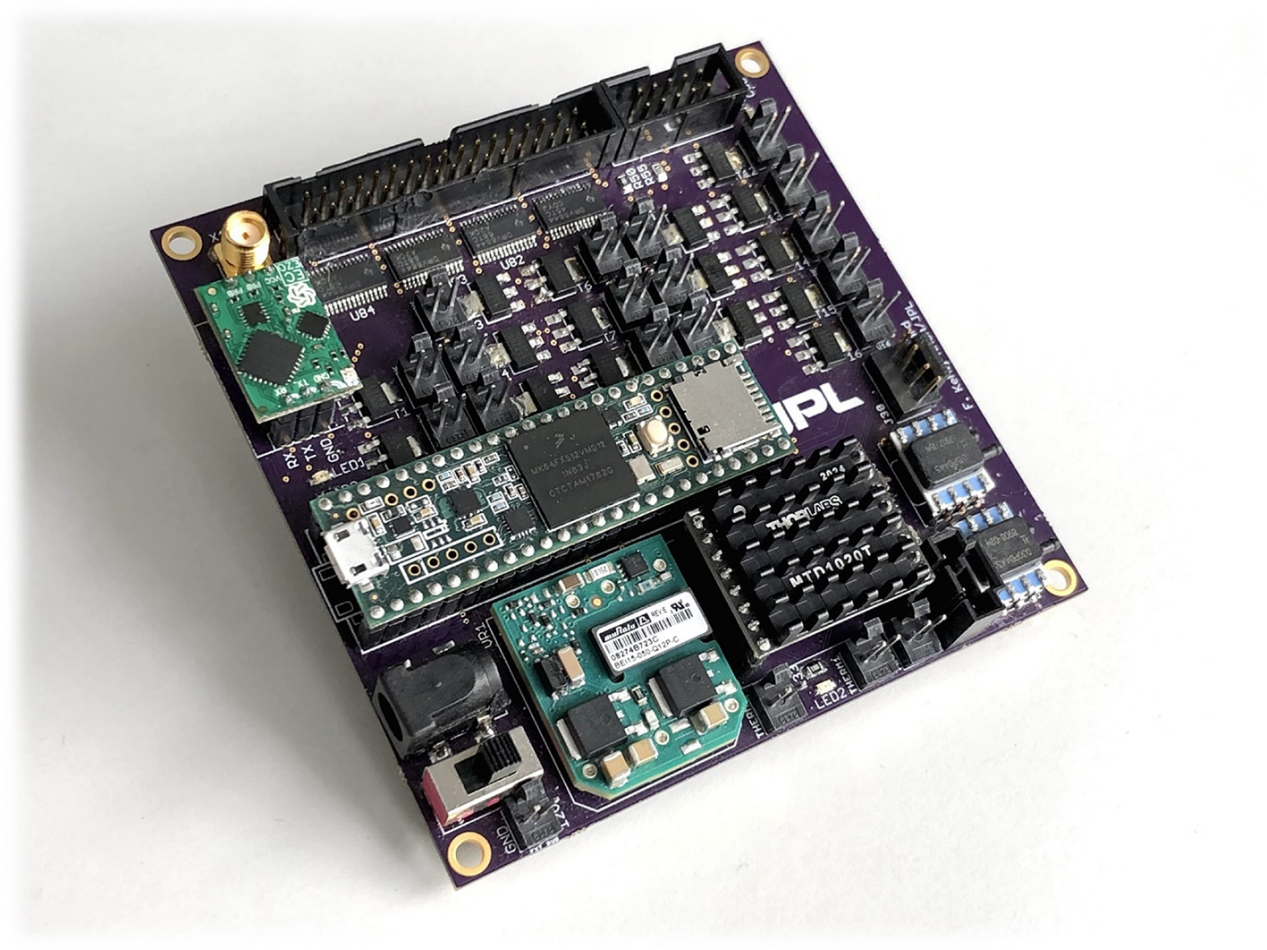
Photograph and summarized capabilities of the assembled MCSP printed circuit board.

Here, we present a compact (96.5 mm × 96.5 mm), versatile, open-source platform which could be employed for a wide range of the everyday microfluidic needs (Fig. 1). The microfluidic control and sensor platform (MCSP) can control:

• solenoid valves, pumps, heater, lamps, or other DC loads
• an additional 16 latching valves
• one thermoelectric cooler (TEC) for temperature control
• a pressure controller
• and a high voltage power supply for electrokinetic or electrophoretic applications

Additionally, it enables measurement and data acquisition from the following components:

• two temperature probes
• ambient temperature and relative humidity sensors
• two pressure sensors
• an electrochemical sensor for electrical conductivity. The board is also supports sensors for pH, oxidation–reduction potential (ORP), or ion-selective electrodes (ISE).
• a bubble (air/liquid) detector
• a liquid flow sensor
• voltage and current of an external high voltage power supply
• a current sensor

The hardware can be directly controlled via USB by any serial console or terminal emulator (e.g., Arduino^1^ Serial Monitor), can be automated with serial command/script execution (e.g., PuTTY^2^, or ZOC^3^), or by writing a custom program and graphical user interface using, for example, Python^4^, LabVIEW^5^, or MATLAB^6^. We chose an Arduino and PlatformIO^7^ IDE compatible Teensy 3.5 microcontroller, as it is compact but still provides sufficient computational power and memory, has an abundance of digital and analog pins, and is well-established within the open-source community.

With this versatile, easy to use, and comparatively low-cost microfluidic control and sensor board, we aim at lowering the entry barrier to microfluidics for research groups with a tighter budget all over the world. We provide an OEM platform for scientists with less background in electrical engineering, which can be used for a broad range of microfluidic applications, with no changes required to the hardware. The sequence of operation desired for a specific experimental setup can easily be defined using the documented serial commands. It is not necessary to make use of all the functional elements on the board. The platform will also work even if only a small section (any of the below features described in Sections 2.3 to 2.10) of the full board is used. For users more experienced in electronics, circuit design, and writing firmware, this manuscript aims at facilitating the development of novel, customized control boards by adapting the provided circuit elements and firmware libraries.

## Hardware Description

The electronics board was custom-designed using Eagle PCB (Autodesk, CA, USA) as a 2-layer printed circuit board (PCB), which can be fabricated in any board house or a PCB milling machine. For the PCB presented in this work, we chose OSH Park (Oshpark LLC, OR, USA) as the manufacturer. The individual components are commercial off-the-shelf (COTS), as detailed in the bill of material (BOM), and are directly soldered to the PCB. For people with less soldering experience, there are also companies that offer turn-key solutions and a fully assembled board when provided layout and BOM. With modularity in mind, either the entire board can be populated to have access to all functionalities or just certain portions that are pertinent to a specific application, which can further lower the overall costs for the board. For example, if an application doesn't require temperature control, electrochemical or pressure sensors, these functional elements, and their components don't have to be soldered to the board. The only essential parts for any partial hardware configuration are the elements presented in sections 2.1 and 2.2.

In the following subchapters, the specific hardware elements will be presented in more detail:

### Microcontroller

**Fig. 2:**
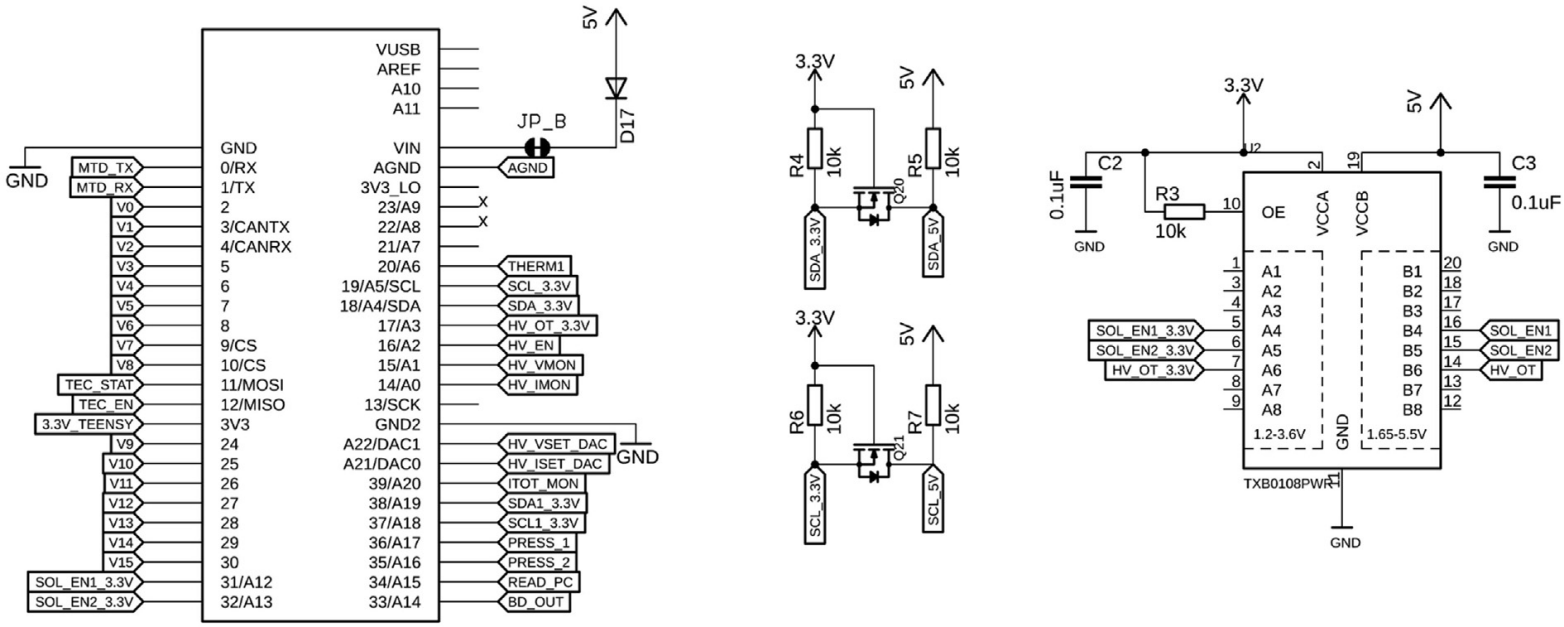
Pin layout of the Teensy 3.5 microcontroller, together with 3.3–5 V level shifters for I^2^C and digital I/Os.

At its core, the board is controlled by a Teensy 3.5 development board (PJRC, CT, USA), a USB-based microcontroller development system. It features a 32-bit 120 MHz ARM Cortex-M4 processor with floating point unit, with 512 and 256 kB flash memory and RAM, respectively. With 58 digital I/O, 27 analog inputs at 13 Bits, two analog outputs at 12 Bits, 20 pulse-width-modulated outputs, various communication protocols/bus systems such as USB, Serial, I^2^C, SPI and CAN bus, and an SD card, it is well equipped to control and read from a multitude of actuators and sensors (Fig. 2). All programming is done via the USB port, and the platform is compatible with the Arduino software and its libraries. The source code for this project, including all the required libraries, are made available in the supplementary materials. The Teensy can be powered directly through the USB cable, but here we recommend powering the Teensy via its VIN pin by applying 5 V, provided directly by the board. To avoid competition between the voltage from the USB and the voltage provided by the board through an external power supply, the VUSB line needs to be cut, which is explained in *Section 5, Build Instructions*.

The Teensy 3.5 also has a slot for a microSD card, which not only enables local data storage but also execution of programs and scripts directly from a file stored on the card. Since the potential application space of this hardware is vast, the users will have to adapt the firmware and implement this feature accordingly, and will hence not be covered in this manuscript.

### Voltage Converters and Current Measurement

**Fig. 3:**
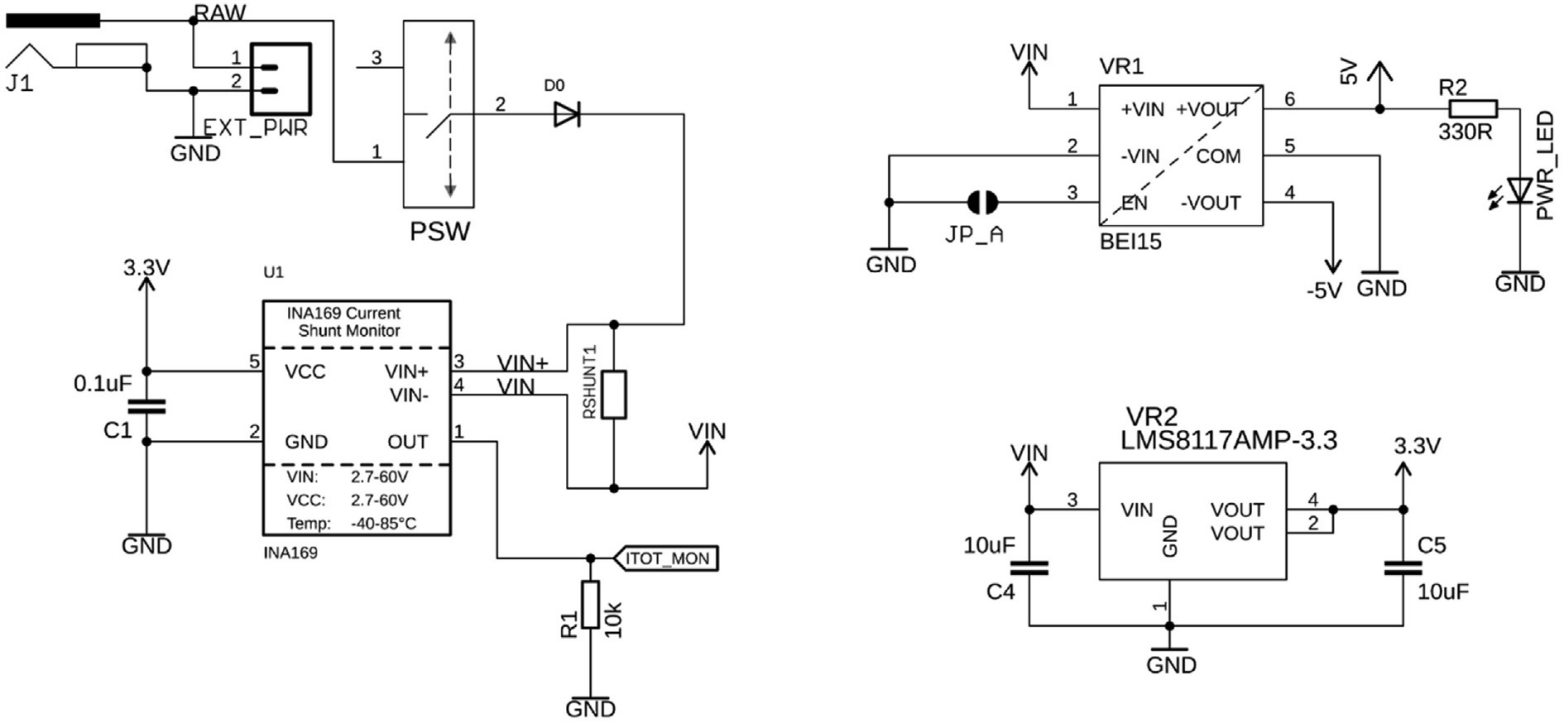
External 12 V power is supplied through J1 or alternatively the EXT_PWR pin header. Two voltage regulators convert the input voltage to + 5 V and −5 V (VR1), and 3.3 V (VR2). The overall consumed current (and hence power) can be measured with U1.

The board is powered via a 5.5 × 2.1 mm barrel connector (J1) or a 2-pin header (EXT_PWR) (Fig. 3). We recommend applying 12 VDC, e.g., by connecting a wall adapter with sufficient power such as the Qualtek QFWB-65–12-US01, which can provide up to 5 A at 12 VDC. The input voltage is fed into two voltage regulators (VR1, VR2) to convert the 12 VDC to voltage rails of 5, 3.3, and −5 VDC. The voltage regulator for the 3.3 VDC rail (LMS8117, Texas Instruments) can provide a maximum current of 1 A, whereas the −5 and 5 VDC regulator (BEI15, Murata Power Solutions, UK) outputs up to 1.5 A per rail. The power to the board can be cut by a sliding switch (PSW), its state indicated by PWR_LED. The board's overall current can be measured by an INA169 current monitor chip, which can provide useful telemetry about the system and overall current (and hence also power) consumption of the peripherals. If no current sensing is required, U1, C1, and R1 can be omitted and are therefore not essential. RSHUNT1 can be replaced with a 0 Ω resistor or the contacts can be bridged by other means. It is recommended that the user comments out the initialization and loop function pair in the firmware top page to stop querying the part which was omitted, in this case the INA169.

### Load Switches for Valves, Pumps, Heaters, Actuators

**Fig. 4:**
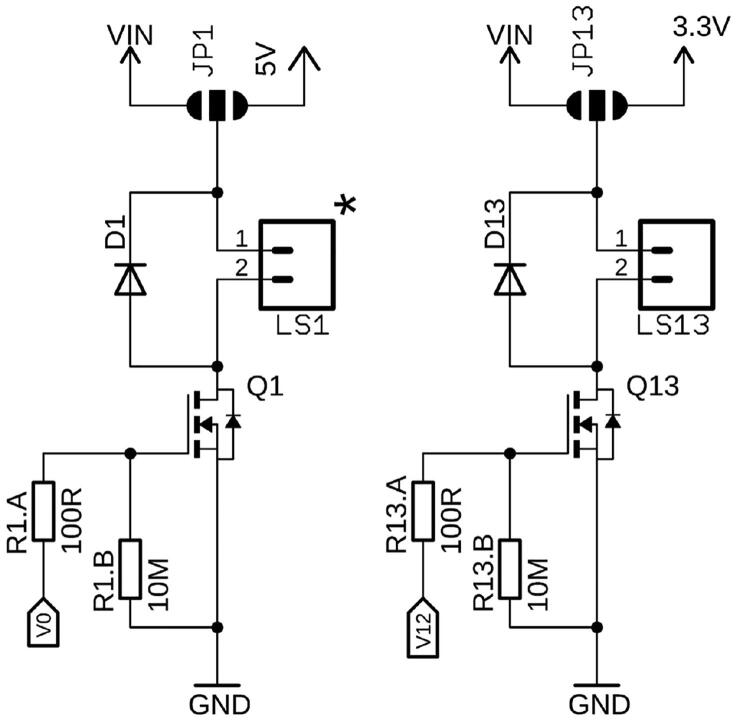
Two of 16 load switches. Load Switch 1 (LS1) can either be supplied with 12 V (VIN) or 5 V, selected by solder jumper JP1. LS1 is a PWM-capable port, as indicated by the asterisk. LS13 can supply either 12 V or 3.3 V, again defined by JP13, and is not a PWM output.

A series of 16 N-channel MOSFET low-side load switches allow independent control of up to 16 components, such as DC motors, solenoids, pumps, valves, resistive heaters, etc., which can be connected to LS1 to LS16. The switches are equipped with a flyback diode, and 11 out of the 16 can be pulse-width modulated (PWM), marked with an asterisk, and are directly controlled via the Teensy's digital I/Os. Solder-jumpers on the back of the board (JP1 – JP16) allow selection between VIN (12 VDC) or 5 VDC for switches 1 to 12, and between VIN (12 VDC) or 3.3 VDC for switches 13 to 16, respectively, depending on what the end-user decides on connecting to the pin header. One instance of both variants (Load Switches LS1 and LS13) is depicted in Fig. 4. Examples of components with relevance in microfluidic applications which can be controlled by these switches include DC motor-driven diaphragm or peristaltic pumps (e.g., NMP series from KNF or RP-Q series from Takasago), solenoid-actuated fixed volume dispense pumps (e.g., LP series, The Lee Co., CT, USA), or non-latching solenoid valves (e.g., LHD or LFN series from The Lee Co, Series 99 valves from Parker, OH, USA, or the NV series from Takasago, Japan). The PWM ports can be used as quasi-analog outputs to control the speed of a DC motor or a pump. They also allow actuation of so-called spike & hold valves, which are advantageous over variants which must be energized at 100% duty cycle. Not only do spike & hold valves provide reduced energy consumption, they also avoid valve damage resulting when valves are kept fully energized at 100% duty-cycle, such as the VHS series micro-dispensing valves from Lee Co. Both spike duration and hold voltage (PWM duty-cycle) can be controlled by a serial command. Also, if desired, the PWM frequency can easily be changed in the firmware settings. We recommend selecting frequencies beyond the audible range to avoid humming sounds of the solenoids. One should be aware that the PWM resolution is inversely proportional to the frequency, such as 3,750,000 Hz at 4 bits, but only 915.527 Hz at 16 bits^8^. For the provided firmware, the authors chose an 8-bit PWM resolution, with a frequency of 234,375 Hz.

### Latching Valves

**Fig. 5:**
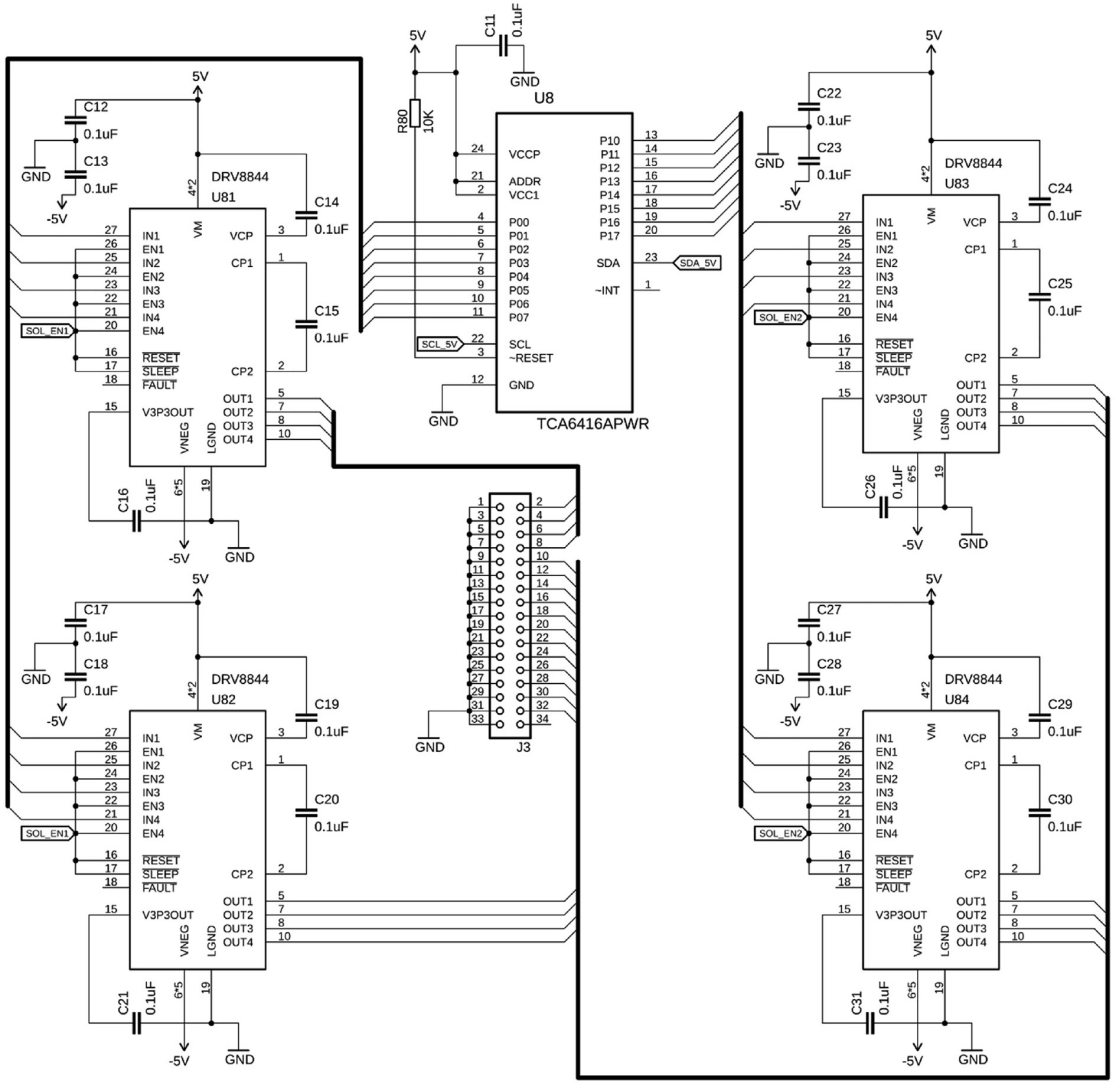
Schematics of the latching valve circuit. A port expander (U8) controls four quadruple ½-H bridges (U81 – U84). Depending on the input and the enable state, the output of the bridges (J3) can be toggled between −5 V, OFF, and +5 V.

Latching valves for fluidics or pneumatics, such as the LHL series from The Lee Co or NLV series from Takasago, are preferred for many applications, because they minimize power consumption and heat generation. The solenoid of these valves is latched by a permanent magnet, maintaining the valve in its current position even if de-energized. The valve is then toggled by inverting the applied potential, and hence current, to the solenoid pins, which is commonly accomplished by an H-bridge circuit. Here, we present an alternative, compact approach using a ½-H-bridge driver (DRV8844, Texas Instruments, TX, USA), which allows simultaneous and independent actuation up to four latching valves per chip. One valve pin is permanently grounded, while the applied potential on the other pin is either +5 VDC (to actuate the valve in the one direction) and −5 VDC (to toggle in the opposite direction). Whether the output of the DRV8844 is positive or negative depends on whether the inputs are HIGH or LOW. The driver chip is also equipped with independent Enable lines. If the Enable line is HIGH, the output is either +5 or −5 VDC, depending on the state of the Input line (hence the valve solenoid is energized). If the Enable line is LOW, the output is left floating, and therefore the valve is de-energized (see Table 1). The presented design (Fig. 5), as implemented on the board, allows actuating up to 16 latching valves (as well as any additional valves connected to the aforementioned Load Switches). Four DRV8844 are controlled by the 16 digital outputs of an I^2^C port expander (TCA6416, Texas Instruments). The Enable lines are bundled into two sets and directly controlled by the Teensy. To actuate a specific set of valves, the Input lines of the valves to toggle are inverted, and the Enable line is pulled HIGH for a short amount of time, usually 10–100 ms, causing the valves to switch their state before it returns to a floating and hence de-energized state (Fig. 16). Due to the voltage rating limitations of the DRV8844, only 5 VDC latching valves can be actuated with this circuit design. The valves can individually be connected to connector J3 by individual jumper wires or multiple valves by a ribbon cable. In the presented design, two Enable lines control eight valves at once to save I/O pins, but if the individual input signal is kept unchanged, the valve position will remain unchanged even if energized for the duration of the Enable pulse.

**Table 1:** Logic table for DRV8844 ½ H-bridge driver.

**Input**	**Enable**	**Resulting Output**
LOW	LOW	Floating
HIGH	LOW	Floating
LOW	HIGH	−5 VDC
HIGH	HIGH	+5 VDC

### Pressure Controller and Sensors

**Fig. 6:**
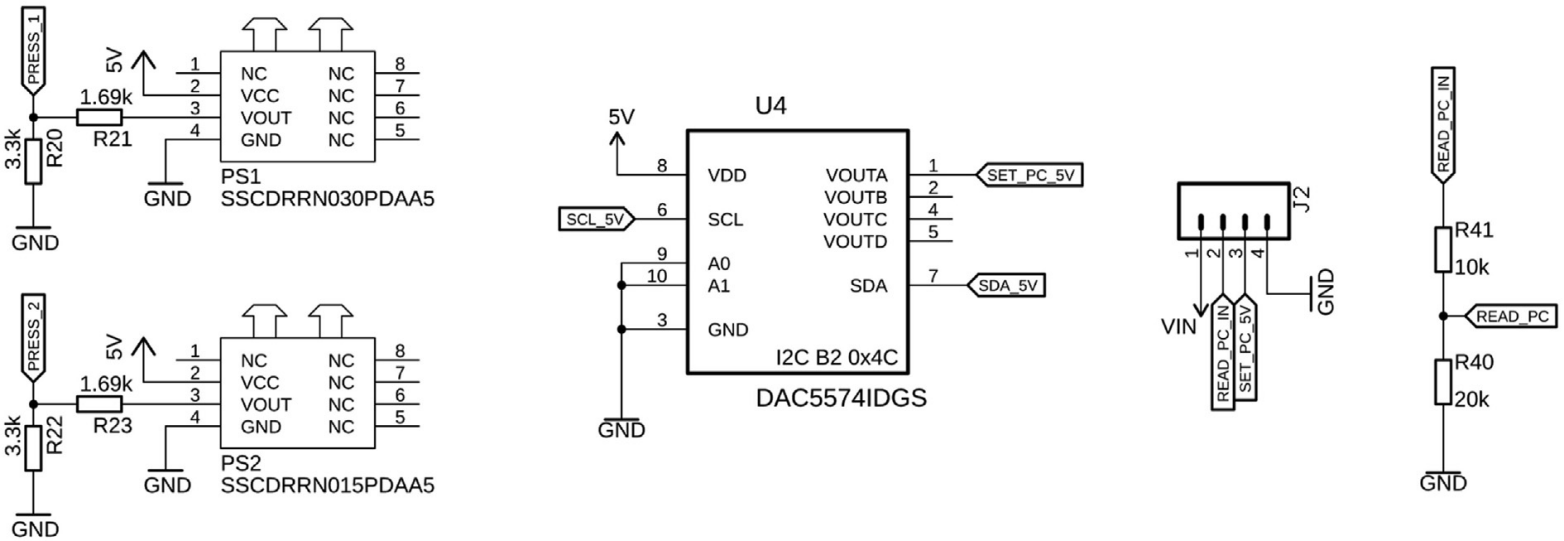
The board is equipped with two analog pressure sensors (PS1, PS2), and an interface to control an external, electronic pressure regulator via J2. The control voltage (SET_PC_5V, Pin 3 on J2) is provided by a digital-to-analog converter (U4), and the internal feedback pressure (READ_PC_IN, Pin2 on J2) can be read by the microcontroller's internal analog-to-digital converter via a voltage divider.

Many microfluidic applications are directly or indirectly controlled by pneumatics, such as using positive or negative pressure to push or pull liquids in a controlled fashion through microfluidic devices or to actuate pneumatic microvalves [7], [8]. The board can power and control an electronic pressure regulator, such as the model 415 from Parker (Parker Hannifin Corporation, PA, USA) (Fig. 6). Via a digital-to-analog converter, the user can control the set pressure between 0 and 5 psi (0 to 0.34 bar) or up to 0–100 psi (0 – 6.9 bar), depending on the model, with a pressure regulation accuracy of ± 1.5% and repeatability of ± 0.25%. The internally measured pressure can be read back via an analog pin. Additionally, two differential pressure sensors (various pressure ranges available) allow precise monitoring of these applied pressures via the microcontroller's analog inputs. The sensors' 5 V maximum output voltages are limited to 3.3 V by voltage dividers to account for the voltage range of the analog inputs. The pressure sensors can easily be connected by their barbed ends to rubber tubing.

### Bubble Detector

It is often very useful for many automated fluidics processing systems to have liquid/gas interface sensors (often referred to as liquid or bubble detectors), be it to count or detect bubbles or droplets, which could, for example, automatically be diverted by valves upon their detection. Other applications include monitoring whether a specific reservoir runs dry to stop a pump or the entire routine automatically, or to check whether a system has sufficiently been primed with fluid (or the opposite, i.e. purged with gas). In combination with a precise, metered pump or known flow-rate, it is even possible to estimate the passing liquid volume in case gas bubbles separate it.

**Fig. 7:**
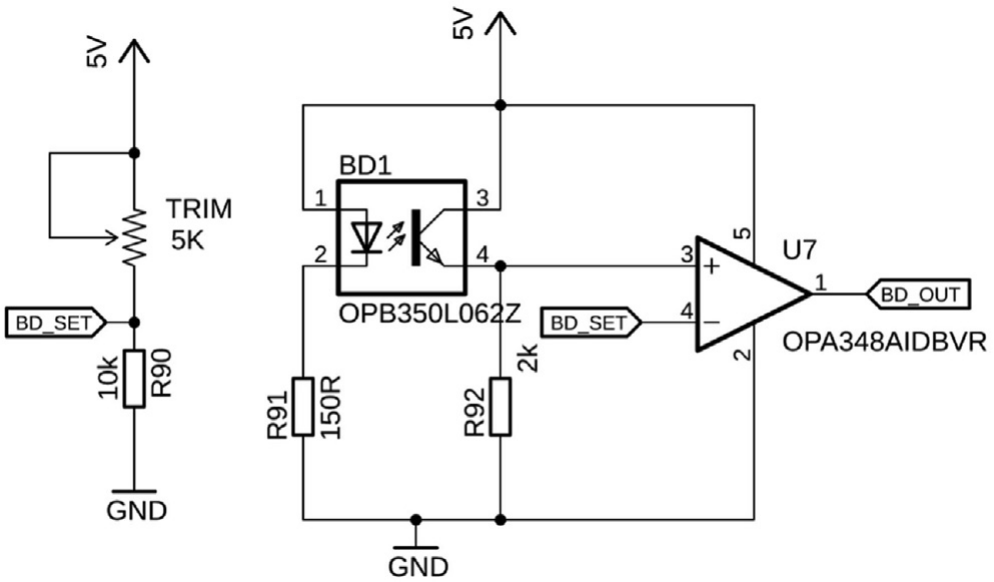
The bubble detector is connected to BD1. Depending on the fluid and tubing, the detection threshold needs to be adjusted by the potentiometer (TRIM).

In this implementation, the board has been designed to accept one OCB350L liquid sensor (TT Electronics, UK), which can be connected to connector BD1 (Fig. 7). These sensors accept 1/16″, 1/8″, 3/16″, and 1/4″ tubing, depending on their exact part number. The optical sensor will register whether there is liquid or gas in the tubing and relay it to the Teensy. Based on the nature of the liquid, its color, turbidity, or refractive index, as well as the transparency of the tubing, the detection threshold can be adjusted with the potentiometer (TRIM).

### High Voltage Power Supply Interface

**Fig. 8:**
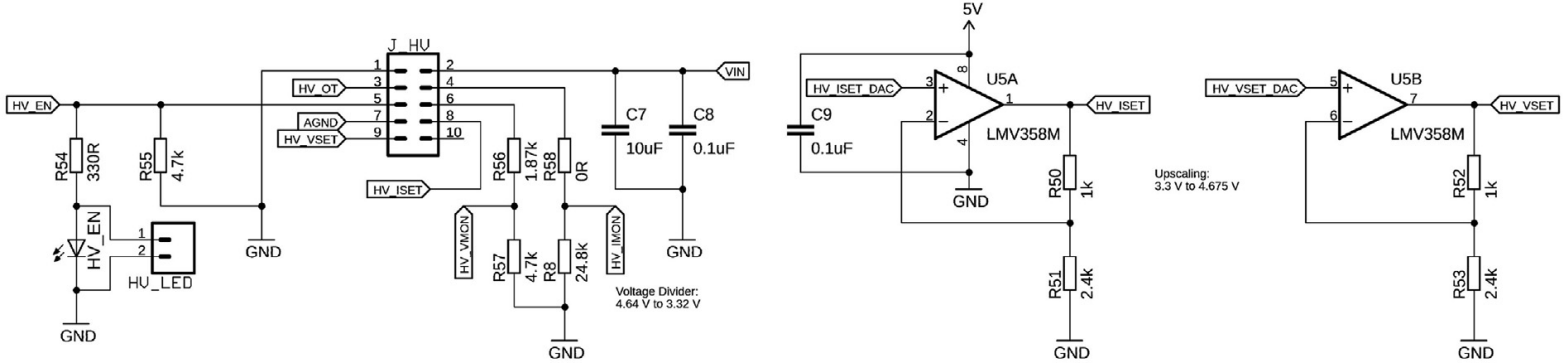
High voltage interface J_HV with the following pin assignments: 1: GND, 2: 12 V power, 3: Over-temperature flag, 4: HV current monitor, 5: Enable line, 6: HV voltage monitor, 7: analog GND, 8: Set HV max. current, 9: Set HV voltage. The VSET and ISET are upscaled to 4.675 V by a 2-channel operation amplifier (U5) to cover the full range of the HVPS. An onboard LED (HV_EN) indicates whether the HVPS is enabled or disabled. Alternatively, an external LED can be connected to HV_LED.

This board was specifically designed for the development of a capillary electrophoresis (CE) system, where high voltages are required to separate charged species. If it is the reader’s intent to build a custom CE system, especially CE coupled to laser-induced fluorescence detection (CE-LIF), the authors would like to refer to two other parts of the series “Open-Source Lab Hardware” [9], [10]. However, not only CE, but a range of microfluidic applications require high voltages, such as electrochromatography, electrokinetic injections, isotachophoresis, electrowetting, and more [11], [12], [13], [14]. The electronics described here are not capable of providing any high voltage (HV) output directly, but rather to interface with an auxiliary high voltage power supply (HVPS) through connector J_HV (Fig. 8). The interface is compatible with Spellman's UM8-40 series DC-DC HVPS (Spellman Corp., Hauppauge, NY), covering voltage ranges from 0 to (-)40 kV and output powers up to 30 W. Through this interface, the output voltage can be set to a specific value. Additionally, the maximum output current of the HVPS can be set by the controller, and while both the actual output voltage and current can be read back via two analog return signals. For safety precautions, the HVPS can be armed or disarmed by an enable line, indicated by an onboard LED (or also an external LED, e.g., for chassis mount, if connected to HV_LED).

### TEC Controller, Thermistor, and Ambient Relative Humidity & Temperature Sensors

**Fig. 9:**
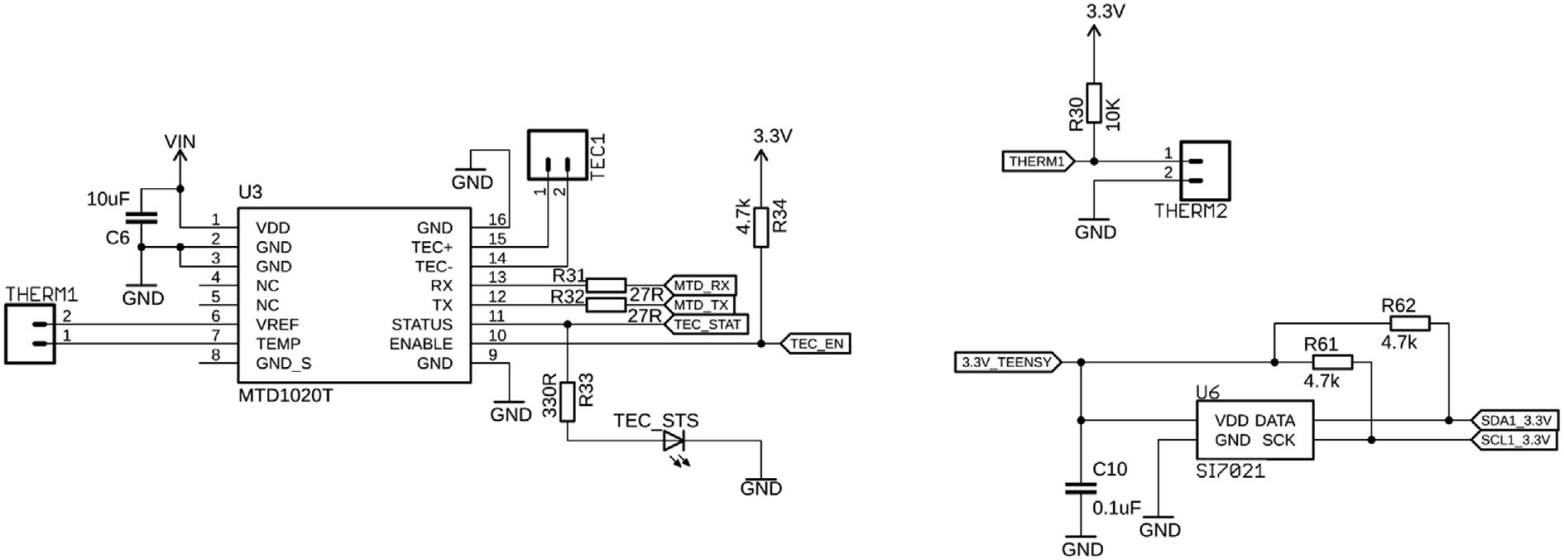
A Peltier element can be connected to TEC1 and controlled by U3. The temperature is monitored with a thermistor connected to THERM1. The TEC status is indicated by an LED (TEC_STS). An additional thermistor can be connected to THERM2 to independently measure an additional temperature. Ambient temperature and relative humidity are measured by U6.

For precise fluid handling, controlled chemical reactions, or accurate sensor readings, it is often crucial to either keep a specific part, or the entire system itself, at a stable and well-defined temperature. This can be accomplished by Peltier elements, which can both actively cool or heat. On this board, we chose to implement an MTD1020T thermo-electric cooler (TEC) controller (Thorlabs, NJ, USA), with a cooling/heating capacity of up to 20 W (Fig. 9). It is mounted to the board by pin headers and controlled by the Teensy via UART. A Peltier element is connected to the TEC1 pin header, whereas the feedback thermistor, taking the temperature of the thermal mass being controlled, is connected to THERM1. The controller can be enabled or disabled by the Teensy via a digital pin, and its status can be read by a second pin and visualized by an LED (TEC_STS). One more thermistor can be connected to the connector THERM2, to take a second, independent measurement at a different location in the system. For specific commands and configuration of the TEC controller, the reader is kindly referred to Thorlab’s MTD1020T manual.

To additionally monitor the ambient relative humidity and temperature, the board is equipped with a Si7021 sensor chip (Silicon Labs, TX, USA), which communicates via the I^2^C lines with the microcontroller directly. Keeping track of these key environmental factors is often desired for the reproducibility of experimental data and vital for lab automation.

### Electrochemical Sensors

**Fig. 10:**
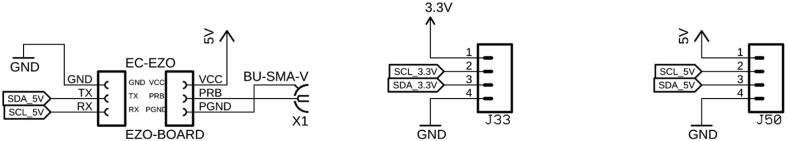
Electrochemical sensors can be connected to the SMA connector X1, while peripheral, additional I^2^C compatible sensors or controllers can be connected to J33 or J50, depending on their respective voltage rating.

To measure pH, oxidation–reduction-potential (ORP), electrical conductivity (EC) of the studied liquids, the board also offers pin headers to accept one EZO^TM^ circuit from Atlas Scientific (Atlas Scientific LCC, NY, USA). Depending on the mounted circuit and the corresponding probe connected to the SMA connector X1 (Fig. 10), the board is capable of measuring either pH, ORP, and EC, as well as dissolved oxygen, flow, and temperature. With the ORP circuit, it is even possible to read from commercial ion-selective electrodes (ISE) to the probe connector. For microfluidic applications, the mini ISEs from NT Sensors (NT Sensors, S.L., Spain) or the ET1601 ISEs from eDAQ (eDAQ Pty Ltd., Australia) might be interesting candidates, as well as the miniaturized flow through electrodes for pH and conductivity, ET916 and ET044, both from eDAQ. Proper grounding and referencing via the probe ground (PGND) with a reference electrode is required if the user chooses to use a miniaturized half-cell electrode. The EZO^TM^ circuits communicate via I^2^C with the Teensy and are controlled via the provided firmware. The user is referred to the EZO^TM^ manual on how to use and calibrate the circuits appropriately.

### Auxiliary I^2^C Ports

The board features two I^2^C pin headers, one for devices with a bus voltage of 3.3 VDC, and one for 5 VDC (Fig. 10). This enables connection of additional, auxiliary devices to the board, enabling it to be tailored to specific applications. Via these auxiliary ports, it is possible, for example, to add additional electrochemical sensor EZO^TM^ boards if multiple parameters need to be monitored at the same time (i.e. temperature and pressure). These ports can also support any other I^2^C compatible device such as LED drivers, displays, non-volatile memory, optical sensors, etc.

For many microfluidic applications, having a precise, in-line flow-rate sensor can be particularly useful. To this end, we wrote libraries that can read from a variety of different, bi-directional liquid flow meters from Sensirion (LG16 and LPG10 series), covering flow ranges from 0.01 to 10,000 µL/min. The flow sensors can directly be connected to the board's auxiliary ports (J33 or J50, depending on voltage) and be monitored by the firmware via the Teensy. To enable operation of the flow sensor, uncomment line 51 in the *void setup tab* on the front page of the firmware, then re-upload the firmware to the board. Conversely, if a flow sensor is not implemented, line 51 needs to be commented out of the code, and then the new code should be uploaded. The LG16 flow sensor is a 5 V device and would therefore have to be connected to J50. The default unit for the flow-rate output is set to nL/min.

**Fig. 11:**
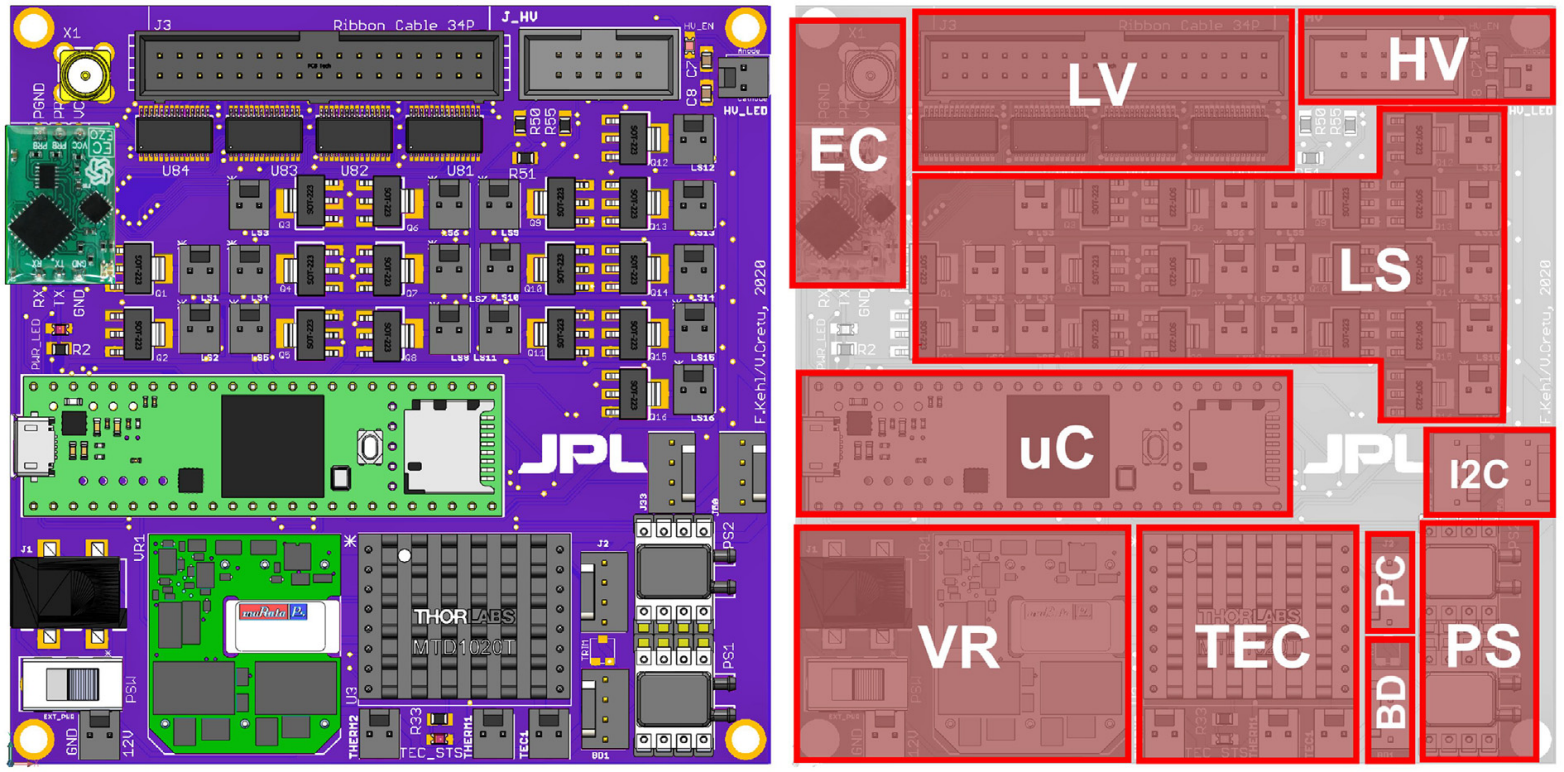
CAD top view of assembled board (left), together with map of the board, highlighting the different hardware elements. EC: Electrochemical Sensor, LV: Latching Valves, HV: HV: High-Voltage Interface, LS: Load Switches, uC: Microcontroller, I2C: I2C Interfaces, VR: Voltage Regulators, TEC: Thermoelectric Controller, PC: Pressure Controller Interface, BD: Bubble Detector Interface, PS: Pressure Sensors.

**Fig. 12:**
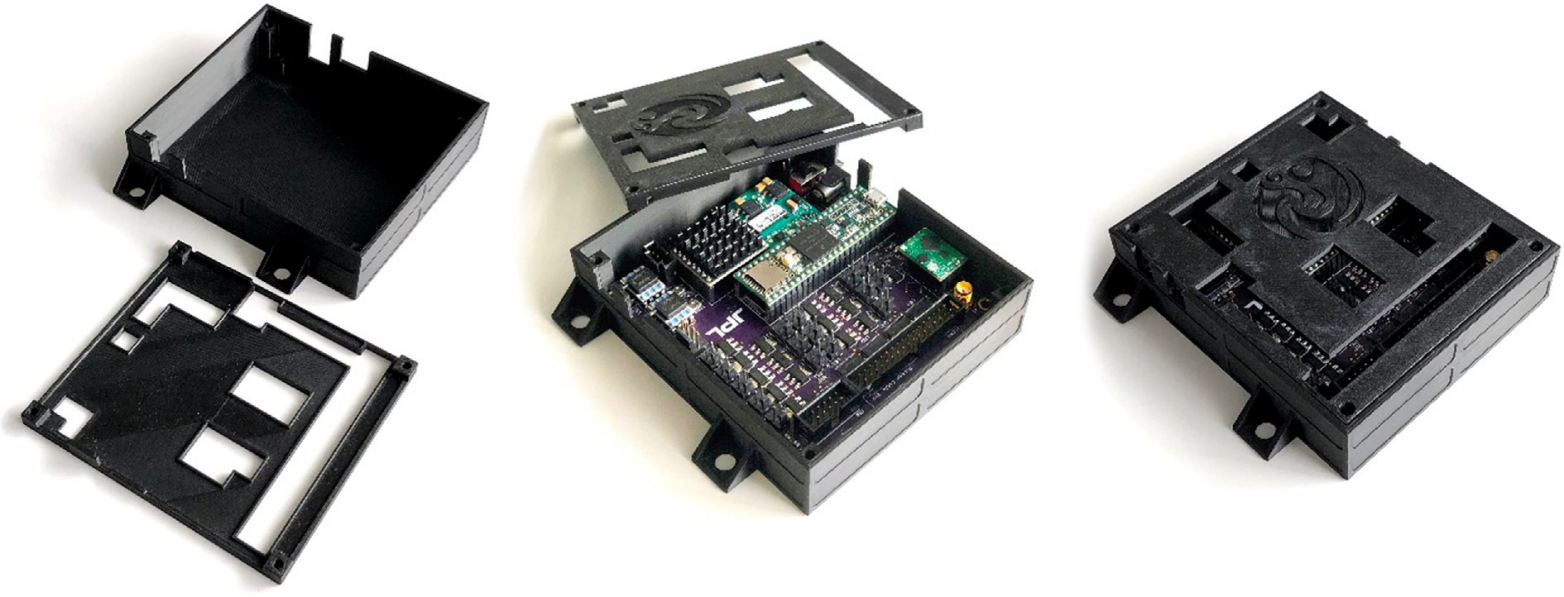
3D-printed enclosure for the board, consisting of a bottom part and a lid.

A CAD rendering of the assembled board, together with the component location is provided in Fig. 11. For convenience, a 3D-printable enclosure is also provided along with the rest of the design files (Fig. 12).

## Design Files

**Table:** 

**Design file name**	**File type**	**Open source license**	**Location of the file**
MCSP PCB V1.0.sch	Schematics	Elsevier User License	https://osf.io/vwhg4/
MCSP PCB V1.0.brd	Layout	Elsevier User License	https://osf.io/vwhg4/
MCSP PCB Libraries V1.0.zip	Library	Elsevier User License	https://osf.io/vwhg4/
MCSP_Firmware_V1.0.zip	Firmware	Elsevier User License	https://osf.io/vwhg4/
MCSP PCBA Parasolid.x_t	CAD	Elsevier User License	https://osf.io/vwhg4/
MCSP Enclosure Box.stl	CAD, 3D-printing	Elsevier User License	https://osf.io/vwhg4/
MCSP Enclosure Lid.stl	CAD, 3D-printing	Elsevier User License	https://osf.io/vwhg4/

**Table:** 

**File**	**Description**
MCSP PCB V1.0.sch	Autodesk Eagle schematic file. The PCB schematic can be viewed and edited if desired.
MCSP PCB V1.0.brd	Autodesk Eagle board file. Needed to produce the PCB.
MCSP PCB Libraries V1.0.zip	Contains required Autodesk Eagle part libraries for the PCB.
MCSP_Firmware_V1.0.zip	Contains the *.ino, *.cpp, and *.h files to program the Teensy controller.
MCSP PCBA Parasolid.x_t	3D model of the PCB assembly.
MCSP Enclosure Box.stl	STL-File to 3D-print the enclosure box.
MCSP Enclosure Lid.stl	STL-File to 3D-print the enclosure lid.

## Bill of Materials

The main supplier of parts for this board is Digi-Key. There are additional suppliers for specific parts such as Atlas Scientifc, Sparkfun, and Thorlabs. Most of the components are 0805 supply package resistors and capacitors, as they do not take up too much board space, yet they are large enough to be easily soldered by hand. The additional integrated circuits were chosen with the same goal in mind, to easily be soldered by hand, hence IC’s with underside pins were not used. Surface mount components were chosen to allow for double-sided placement, which drastically reduces the size of the board and keep the overall cost down. The bill of materials is presented below.

PCB Board:

**Table:** 

**Designator**	**Component**	**Number**	**Cost per unit - USD**	**Total cost -USD**	**Supplier**	**Supplier Part. No.**	**Manufacturer Part. No.**

BD1	SENSOR OPT SLOT PHOTOTRANS MODUL	1	7.63	7.63	Digi-Key	365–1406-ND	OPB350W062Z
C1, C2, C3, C8, C9, C10, C11, C12, C13, C14, C15, C16, C17, C18, C19, C20, C21, C22, C23, C24, C25, C26, C27, C28, C29, C30, C31	CAP CER 0.1UF 50 V X7R 0805	27	0.07	1.97	Digi-Key	478–10836-1-ND	08055C104KAT4A
C4, C5, C6, C7	CAP CER 10UF 25 V X5R 0805	4	0.2	0.80	Digi-Key	1276–6454-1-ND	CL21A106KAYNNNG
D0	DIODE SCHOTTKY 40 V 2A DO214AC	1	0.41	0.41	Digi-Key	641–1696-1-ND	CDBA240-HF
D1, D2, D3, D4, D5, D6, D7, D8, D9, D10, D11, D12, D13, D14, D1, D16, D17	DIODE SCHOTTKY 40 V 1A SMB	17	0.30	5.13	Digi-Key	MBRS140FSCT-ND	MBRS140
EC-EZO	EZO™ Conductivity Circuit	1	59.99	59.99	Atlas Scientific	EZO-EC	EZO-EC
EC-EZO HEADER	CONN SOCKET 3POS 0.1 GOLD PCB	2	0.4	0.80	Digi-Key	1212–1180-ND	801–87-003–10-001101
EXT_PWR, HV_LED, LS1, LS2, LS3, LS4, LS5, LS6, LS7, LS8, LS9, LS10, LS11, LS12, LS13, LS14, S15, LS16, TEC1, THERM1, THERM2	CONN HEADER VERT 2POS 2.54MM	21	0.11	2.25	Digi-Key	A1921-ND	640456–2
HV_LED	LED RED CLEAR 0805 SMD	1	0.18	0.18	Digi-Key	732–4985-1-ND	150080SS75000
J_HV	CONN HEADER VERT 10POS 2.54MM	1	0.28	0.28	Digi-Key	ED1543-ND	302-S101
J1	DC Barrel Power Jack/Connector (SMD)	1	1.5	1.50	Sparkfun	PRT-12748	ADC-H-028–1
J2, J33, J50	CONN HEADER VERT 4POS 2.54MM	3	0.19	0.57	Digi-Key	A19431-ND	640454–4
J3	CONN HEADER VERT 34POS 2.54MM	1	0.68	0.68	Digi-Key	S9174-ND	SBH11-PBPC-D17-ST-BK
MC	TEENSY 3.5 W/ HDRS K64 EVAL BRD	1	31.25	31.25	Digi-Key	1568–1464-ND	DEV-14056
PCB	Custom	1	24.08	24.08	OSHPark	Custom	Custom
PS1	SENSOR PRESS 30PSI DIFF 5 V DIP	1	26.75	26.75	Digi-Key	480–3826-5-ND	SSCDRRN030PDAA5
PS2	SENSOR PRESS 15PSI DIFF 5 V DIP	1	34.02	34.02	Digi-Key	480–5396-5-ND	SSCDRRN015PDAA5
PSW	SWITCH SLIDE SPDT 6A 120 V	1	4.17	4.17	Digi-Key	CKN5001-ND	1101M2S3CQE2
PWR_LED	LED GREEN CLEAR 0805 SMD	1	0.18	0.18	Digi-Key	732–4986-1-ND	150080VS75000
Q1, Q2, Q3, Q4, Q5, Q6, Q7, Q8, Q9, Q10, Q11, Q12, Q13, Q14, Q15, Q16	MOSFET N-CH 55 V 5.2A SOT223	16	1.57	25.04	Digi-Key	AUIRLL2705TRCT-ND	AUIRLL2705TR
Q20, Q21	MOSFET N-CH 50 V 220MA SOT-23	2	0.26	0.52	Digi-Key	BSS138CT-ND	BSS138
R1, R3, R4, R5, R6, R7, R30, R41, R80, R90	RES SMD 10 K OHM 1% 1/3W 0805	10	0.09	0.89	Digi-Key	A126417CT-ND	CRGH0805F10K
R1.A, R2.A, R3.A, R4.A, R5.A, R6.A, R7.A, R8.A, R9.A, R10.A, R11.A, R12.A, R13.A, R14.A, R15.A, R16.A	RES 100 OHM 1% 1/4W 0805	16	0.07	1.12	Digi-Key	RNCP0805FTD100RCT-ND	RNCP0805FTD100R
R1.B, R2.B, R3.B, R4.B, R5.B, R6.B, R7.B, R8.B, R9.B, R10.B, R11.B, R12.B, R13.B, R14.B, R15.B, R16.B	RES 10 M OHM 1% 1/8W 0805	16	0.03	0.51	Digi-Key	RMCF0805FT10M0CT-ND	RMCF0805FT10M0
R2, R33, R54	RES 330 OHM 1% 1/8W 0805	3	0.1	0.30	Digi-Key	RMCF0805FT330RCT-ND	RMCF0805FT330R
R20, R22	RES 3.3 K OHM 1% 1/8W 0805	2	0.1	0.20	Digi-Key	RMCF0805FT3K30CT-ND	RMCF0805FT3K30
R21, R23	RES SMD 1.69 K OHM 1% 1/8W 0805	2	0.1	0.20	Digi-Key	P1.69KCCT-ND	ERJ-6ENF1691V
R31, R32	RES SMD 27 OHM 1% 1/8W 0805	2	0.1	0.20	Digi-Key	A126357CT-ND	CRG0805F27R
R34, R55, R57, R61, R62	RES 4.7 K OHM 1% 1/8W 0805	5	0.1	0.50	Digi-Key	RMCF0805FT4K70CT-ND	RMCF0805FT4K70
R40	RES 20 K OHM 1% 1/4W 0805	1	0.1	0.10	Digi-Key	RNCP0805FTD20K0CT-ND	RNCP0805FTD20K0
R50, R52	RES SMD 1 K OHM 1% 1/3W 0805	2	0.1	0.20	Digi-Key	A126422CT-ND	CRGH0805F1K0
R51, R53	RES 2.4 K OHM 1% 1/4W 0805	2	0.1	0.20	Digi-Key	2019-RK73H2ATTD2401FCT-ND	RK73H2ATTD2401F
R56	RES SMD 1.87 K OHM 1% 1/8W 0805	1	0.1	0.10	Digi-Key	P1.87KCCT-ND	ERJ-6ENF1871V
R8	RES SMD 24.9 K OHM 1% 1/8W 0805	1	0.1	0.10	Digi-Key	13-RT0805FRE0724K9LCT-ND	RT0805FRE0724K9L
R58	RES SMD 0 OHM 1% 1/8W 0805	1	0.1	0.10	Digi-Key	541–4126-1-ND	CRCW08050000Z0EBC
R91	RES 150 OHM 1% 1/4W 0805	1	0.1	0.10	Digi-Key	RNCP0805FTD150RCT-ND	RNCP0805FTD150R
R92	RES 2 K OHM 1% 1/4W 0805	1	0.1	0.10	Digi-Key	RNCP0805FTD2K00CT-ND	RNCP0805FTD2K00
RSHUNT1	RES 0.1 OHM 1% 7 W 2512	1	1.3	1.30	Digi-Key	511–1692-1-ND	GMR100HTCFAR100
TEC HEADER	CONN SOCKET 8POS 0.1 GOLD PCB	2	0.78	1.56	Digi-Key	1212–1190-ND	801–87-008–10-001101
TEC_STS	LED YELLOW CLEAR 0805 SMD	1	0.18	0.18	Digi-Key	732–4987-1-ND	150080YS75000
TEENSY HEADER	CONN HDR 24POS 0.1 GOLD PCB	2	1.43	2.86	Digi-Key	S7057-ND	PPPC241LFBN-RC
TRIM	TRIMMER 5 K OHM 0.1 W J LEAD TOP	1	0.17	0.17	Digi-Key	TC33X-502ECT-ND	TC33X-2-502E
U1	IC CURRENT MONITOR 0.5% SOT23-5	1	2.51	2.51	Digi-Key	296–26063-1-ND	INA169NA/3K
U2	IC TRNSLTR BIDIRECTIONAL 20TSSOP	1	1.29	1.29	Digi-Key	296–21527-1-ND	TXB0108PWR
U3	TEC Driver, THT Package with Heatsink	1	79.8	79.80	Thorlabs	MTD1020T	MTD1020T
U4	IC DAC 8BIT V-OUT 10VSSOP	1	4.34	4.34	Digi-Key	296–16665-5-ND	DAC5574IDGS
U5	IC OPAMP GP 2 CIRCUIT 8SOIC	1	0.44	0.44	Digi-Key	296–9571-1-ND	LMV358IDR
U6	SENS HUMID/TEMP 3.6 V I2C 3% 6DFN	1	3.06	3.06	Digi-Key	336–4379-1-ND	SI7021-A20-GMR
U7	IC OPAMP GP 1 CIRCUIT SOT23-5	1	0.66	0.66	Digi-Key	296–26277-1-ND	OPA348AIDBVR
U8	IC I/O EXPANDER I2C 16B 24TSSOP	1	1.55	1.55	Digi-Key	296–24536-1-ND	TCA6416APWR
U81, U82, U83, U84	IC MTR DRV BIPOLR 8–60 V 28HTSSOP	4	3.31	13.24	Digi-Key	296–34903-1-ND	DRV8844PWPR
VR1	DC DC CONVERTER +/-5V 15 W	1	32.5	32.50	Digi-Key	811–2181-5-ND	BEI15-050-Q12P-C
VR2	IC REG LINEAR 3.3 V 1A SOT223	1	1.19	1.19	Digi-Key	LMS8117AMP-3.3/NOPBCT-ND	LMS8117AMP-3.3/NOPB
X1	CONN SMA RCPT STR 50 OHM PCB	1	2.97	2.97	Digi-Key	CONSMA001-ND	CONSMA001

Enclosure:

**Table:** 

Designator	Component	Number	Cost per unit - USD	Total cost -USD	Source: McMaster-Carr part number	Material type
Heat Inserts	4–40 Tapered Heat-Set Inserts for Plastic	8	0.1265	1.012	93365A122	Brass
Screws	18–8 Stainless Steel Socket Head Screws	8	0.0481	0.3848	92196A105	Stainless Steel

## Build Instructions

**Fig. 13:**
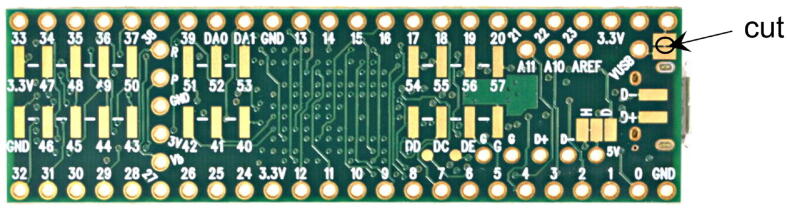
Bottom side of a Teensy 3.5. For nominal operation with the presented board, the indicated trace needs to be cut.
• Although the Teensy controller itself and other components such as the TEC controller or electrochemical sensor board could be directly soldered to the mainboard, the authors recommend using female socket connectors for easy replacement. Make sure to mount these components in the correct orientation.
• Close solder bridges for individual load switches according to the required voltage for specific application (Fig. 14). Connect the middle pad to the left or right pad. Never bridge all three pads since this would cause a short between two power rails. If the solder jumpers J1 – J16 are not set in either configuration, the load switches will not be operational.

PCB Assembly:

• Solder all SMD components to the PCB first, followed by soldering all through-hole components and connectors.
• Cut the trace between the two solder jumpers at the top right on the back of the Teensy to separate VIN from VUSB, to use the power provided by the mainboard (Fig. 13).

**Fig. 14:**
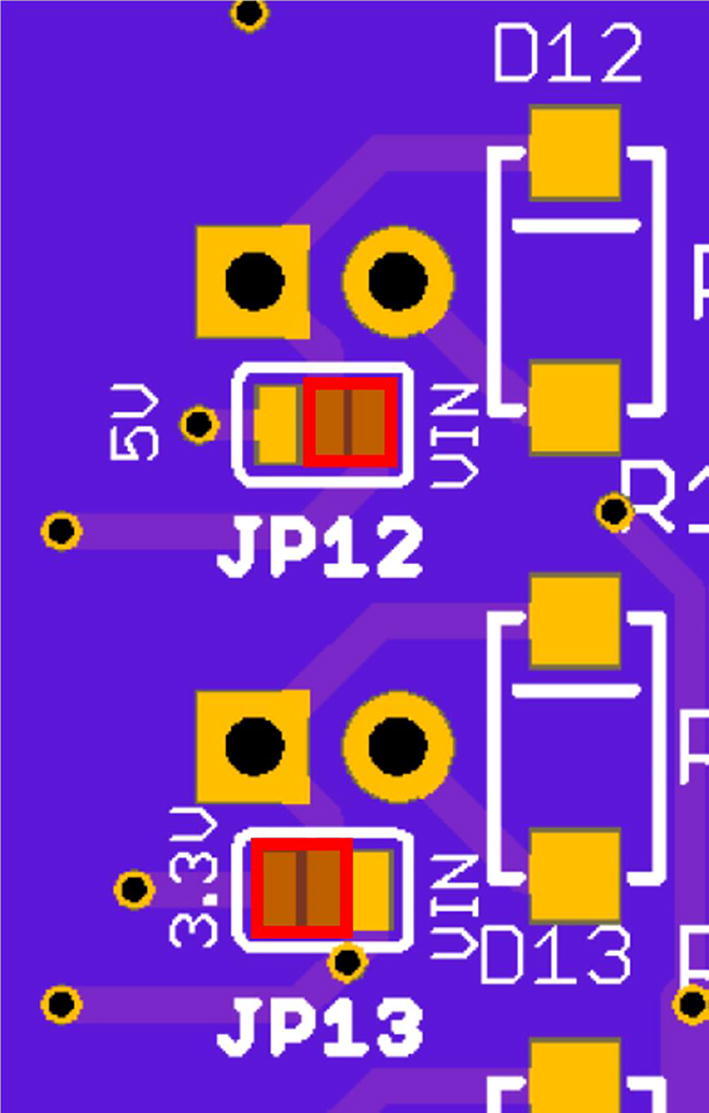
Close solder bridges to select load switch voltage on the back of the board. Here, for example, JP12 is connected to right pad (VIN), and hence 12 VDC would be applied to LS12. For JP13, the central pad is connected to the left pad, therefore 3.3 VDC would be applied to LS13.

Enclosure:

• 3D print enclosure bottom and lid.
• Install 4–40 (or M3) heat inserts or tap screw holes directly.
• Mount board inside the bottom enclosure using four 4–40 (or M3) screws.
• Close lid using four 4–40 (or M3) screws.

## Operation Instructions

### Upload Firmware

In this section, the initial programming of the microcontroller is explained. Unless the user wishes to customize the firmware, this only needs to be done once. If the Teensy controller needs to be replaced, repeat steps 4 – 6.

1. Download and install Arduino IDE^9^ (or similar).
2. Download and install Teensyduino^10^.
3. Download *.zip file containing the firmware (MCSP_Firmware_V1.0.zip) from document repository^11^, extract the zip-file, and open the *.ino file in Arduino, under “File > Open…”.
4. Connect the power supply and USB cable. Make sure the main power switch on the board is in the ON position.
5. In Arduino, go to “Tools > Board” and select Teensy 3.5, and under “Tools > Port” the corresponding COM-port where your Teensy is connected.
6. Go to “Sketch > Upload” to compile the code and program your Teensy controller.

### Direct Control

1. Connect the power supply and USB cable. Make sure the main power switch on the board is in the ON position.
2. Connect the peripherals, such as valves, pumps, and sensors.
3. Establish communication with the board:
  • In Arduino, under “Tools > Port”, select the corresponding COM-port where your Teensy is connected.
  • In Arduino, open Serial Monitor under “Tools > Serial Monitor”.
  • Make sure the Baud Rate is 115,200 and both, NL and CR, are selected.
  • Type individual commands (as specified in the Serial Command Document) in the command line and send by clicking “send” or by pressing “Enter” on the keyboard.
  • Continue by sending the next desired command.

## Serial Command List:

Below, a list of serial commands that can be sent to the microcontroller. Most commands must begin with *{* and end with *}*. Inside the brackets is the command itself. A command consists of a series of identifying characters and a number of arguments separated by spaces. An example command would be { A B 100 1000 }, where *A* and *B* are the identifying characters, *100* the first argument, and *1000* the second argument. It will be listed in the following format:

**Table:** 

**Serial Command**	**Description**

{A B xxx yyyy}	Example command, where e.g., xxx = [0–100], and yyyy = [0–1000].
Hi / hello	System should respond to make sure communication works
Help	Displays all commands and description

**Bubble Detector:**

**Table:** 

{ B }	Reads and prints the number of bubbles that have been detected

{ B x }	Reads and prints the bubble count every second. x = [1/0] (ON/OFF)

**Flow Sensor:**

**Table:** 

{ F }	Reads and prints the measured flow rate in nL/min once

{ F x }	Reads and prints flow rate nL/min every second. x = [1/0] (ON/OFF)

**High Voltage Power Supply:**

**Table:** 

{ H E x }	Enable/Disable HVPS. x = [1/0] (ON/OFF)
{ H }	Reads and prints the HVPS current, voltage, and overtemperature once (in 10-bit integer).
{ H x }	Reads and prints the HVPS current, voltage, and overtemperature every second (in 10-bit integer). x = [1/0] (ON/OFF)
{ H V xxx }	Sets the output voltage of the HVPS to [xxx = 0–255] (in 8-bit integer)*
{ H I xxx }	Sets the max. output current of the HVPS to [xxx = 0–255] (in 8-bit integer)*

*this code was written for Spellman's UM20 HVPS, with a maximum voltage of 20 kV and a maximum output current of 200 uA. This command essentially controls the HV_VSET and HV_ISET pin of the HV interface to set the analog voltage between 0 and 5 V. The actual output depends on the HVPS used.

**Current Sensor:**

**Table:** 

{ i }	Reads and prints the total current in mA
{ i × }	Reads and prints the total current in mA every second. × = [1/0] (ON/OFF)

**Temperature Controller:**

**Table:** 

{ M x }	Enable/Disable MTD1020T controller. x = [1/0] (ON/OFF)
M xx	Send commands to the TEC controller. Refer to the MTD1020T datasheet for the list of commands. Example command: M m? reads and prints the version of hardware and firmware on the MTD1020T.

**Pressure Sensors:**

**Table:** 

{ P }	Reads and prints the measured pressures once (in 10-bit integer)**
{ P x }	Reads and prints the measured pressures every second (in 10-bit integers). x = [1/0] (ON/OFF)**

**pressure range and units can be changed in the firmware (Pressure_Sensors.cpp, lines 24/25 and 37/38), depending on the range of the pressure sensors and the preferred units.

**Load Switches:**

**Table:** 

{ S xx yyy }	xx = switch number [1–16], yyy = [0–255]. NOTE: if y = 0, switch will be OFF, if y = 1, then switch will be on at 100% duty-cycle. Anything in between is pulse-width-modulated if pin is able to.

**Spike and Hold Operation:**

**Table:** 

{ S H xx y t }	xx = switch number (1–9, 15, 16); y = hold voltage (0–255); t = 0 = off, t = 1 = ON indefinitely, t > 1 = Spike duration time in ms.

**Temperature and Humidity Sensor:**

**Table:** 

{ T }	Reads and prints the temperature in degrees C and relative humidity in % once

{ T x }	Reads and prints the temperature in degrees C and relative humidity every second. x = [1/0] (ON/OFF)

**Latching Valves:**

**Table:** 

{ V S xx y }	Latches a single valve. xx = valve number [1 – 16], y = [1/0] (HI/LO)

{ V xxxxxxxx yyyyyyyy }	Latches multiple valves at the same time. xxxxxxxx = MSB, yyyyyyyy = LSB. Example: { V 11,000,000 00,000,001 }, latches valves 1, 15, and 16 HI, all the other valves LO.
{ v }	Latches all valves LO
{ V }	Latches all valves HI

**Pressure Controller**:

**Table:** 

{ P C xxx }	Sets the pressure controller output voltage to [0–5 V] where xxx = 0–255

**Electrochemical Sensor:**

**Table:** 

{ E }	Read and print the EC value once
{ E x }	Read and print the EC value continuously at 1 Hz. x = [1/0] (ON/OFF)
{ E C Q }	View calibration type in memory
{ E C C }	Clear current calibration in memory
{ E C D }	Perform a dry calibration
{ E C S x }	Single point calibration, x = EC value in µS/cm
{ E C L x }	Two point calibration, Low: x = EC value in µS/cm
{ E C H x}	Two point calibration, High: x = EC value in µS/cm
{ E K x }	Probe constant calibration: x = [1, 10, 100]
{ E K Q }	View the current probe constant calibration in memory

The user is referred to the Atlas Scientific EZO^TM^ datasheet for detailed operation and calibration.

The above is a selection of basic commands, allowing the user to explore the possibilities of the presented electronics board. The authors understand that some users might have different requirements and hence encourage them to amend the firmware and command library how they see best fit. Other Atlas Scientific EZO^TM^ sensor boards (such as pH, ORP, DO) can also be used in combination with the presented hardware.

### Automated Control

The hardware can be automated with serial command/script execution (e.g., PuTTY, or ZOC), or by writing a custom program and graphical user interface using, for example, Python, LabVIEW, or MATLAB. The firmware could also be modified in such a way that an automated protocol or state machine could be written as part of the firmware itself, or the sequence of operation could be read from a file on the SD card. Since the different options and applications are vast, this will not be covered in this publication and is considered beyond the scope of this paper. A general description on how to communicate with the SD card is, for example, provided here: https://github.com/greiman/SdFat.

## Validation and Characterization

The potential applications with the presented hardware are countless and it is not possible to cover all of them here. A few basic and general functions are presented below. While the control interface of the thermo-electric controller and the flow sensor to this board have been verified by the authors, a comprehensive demonstration or validation is beyond the scope of this paper, and the reader is referred to the manual of the respective sensors.

### Load Switches

**Fig. 15:**
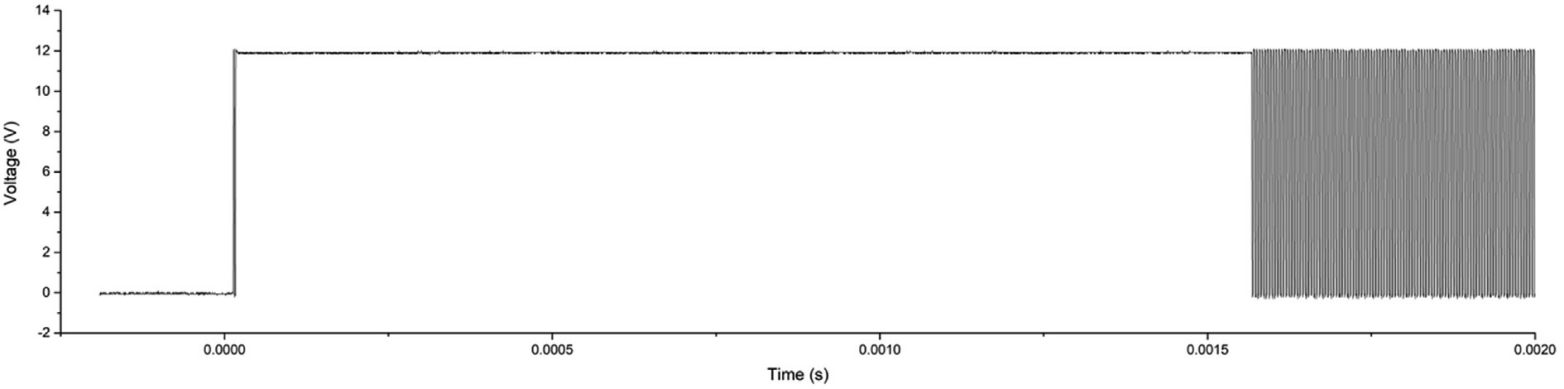
Oscillograph of a spike & hold actuation of a load switch across a 250 Ω resistor. The spike duration was 16 ms at 12 VDC, before switching to a 50% duty-cycle.

Besides switching connected loads, such as valves, pumps, motors, etc. at 3.3, 5, or 12 V, the load switches can be operated in the previously mentioned spike & hold fashion. To do so, the full potential is applied for a brief period of time (“spike”, e.g., to provide sufficient energy to move a valve piston), but then less power is required to “hold” this piston in its position. This is achieved by reducing the duty-cycle of the PWM signal. Fig. 15 depicts a recorded oscillograph across a dummy load (250 Ω resistor) using an HMO1024 oscilloscope (Rohde & Schwarz, Germany), spiking to 12 V and then switching to a duty-cycle of 50%.

These load switches have been verified to work with a series of spike & hold valves (e.g. LFN and VHS series from The Lee Co, Series 99 valves from Parker), pumps both for liquid and air (e.g. RP-Q series from Takasago, LPM and LPL series from The Lee Co., and CTS series from Parker).

### Latching Valves

**Fig. 16:**
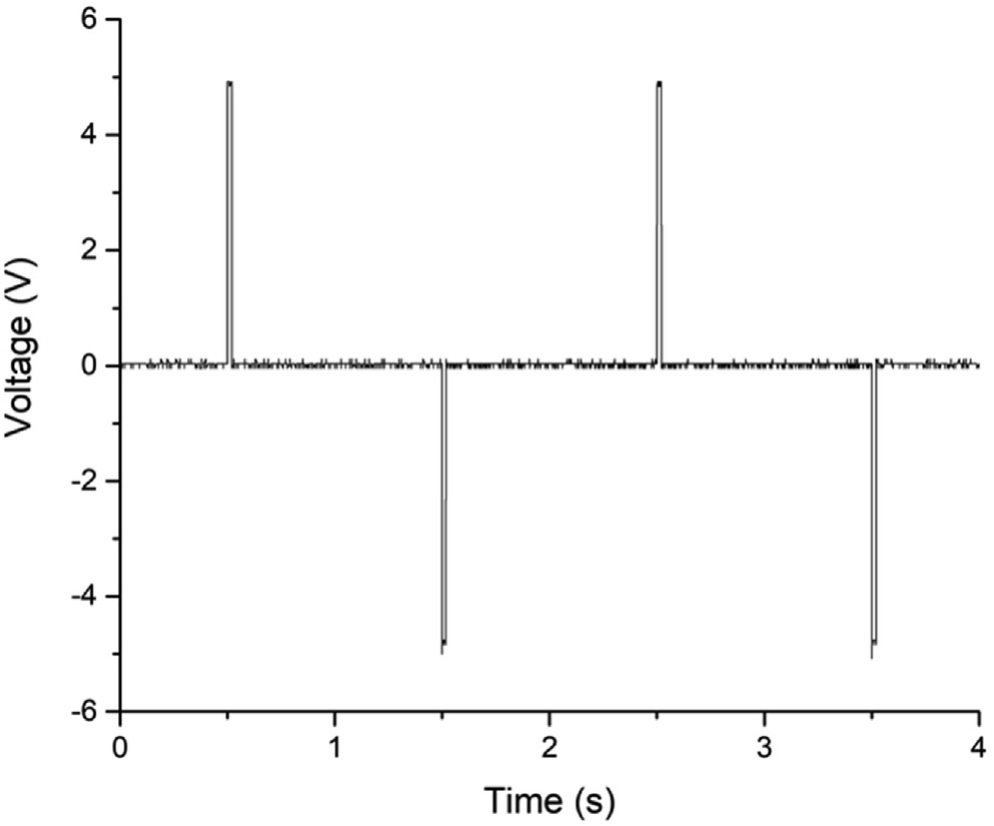
Oscillograph of two latching valve pulses to + 5 V and two to −5 V, each 100 ms long and 1 s in between.

As a permanent magnet keeps latching valves in their current state, no holding current is required, and the valve can be de-energized after an initial spike. This spike allows the valve to switch its state. If the polarity of this spike is reversed, the valve switches to its opposite state, and the valve can be de-energized again. In Fig. 16, a sequence of four spikes is recorded using the abovementioned oscilloscope. A first peak to +5 V for 100 ms is used to actuate the latching valve in one direction, followed by a second spike to −5 V after 1 s, to switch the valve position, and a third spike again at +5 V.

This circuit to control latching valves has been verified with LHL Series 2- and 3-port magnetically latched solenoid valves from The Lee Co.

### Pressure Controller & Sensors

**Fig. 17:**
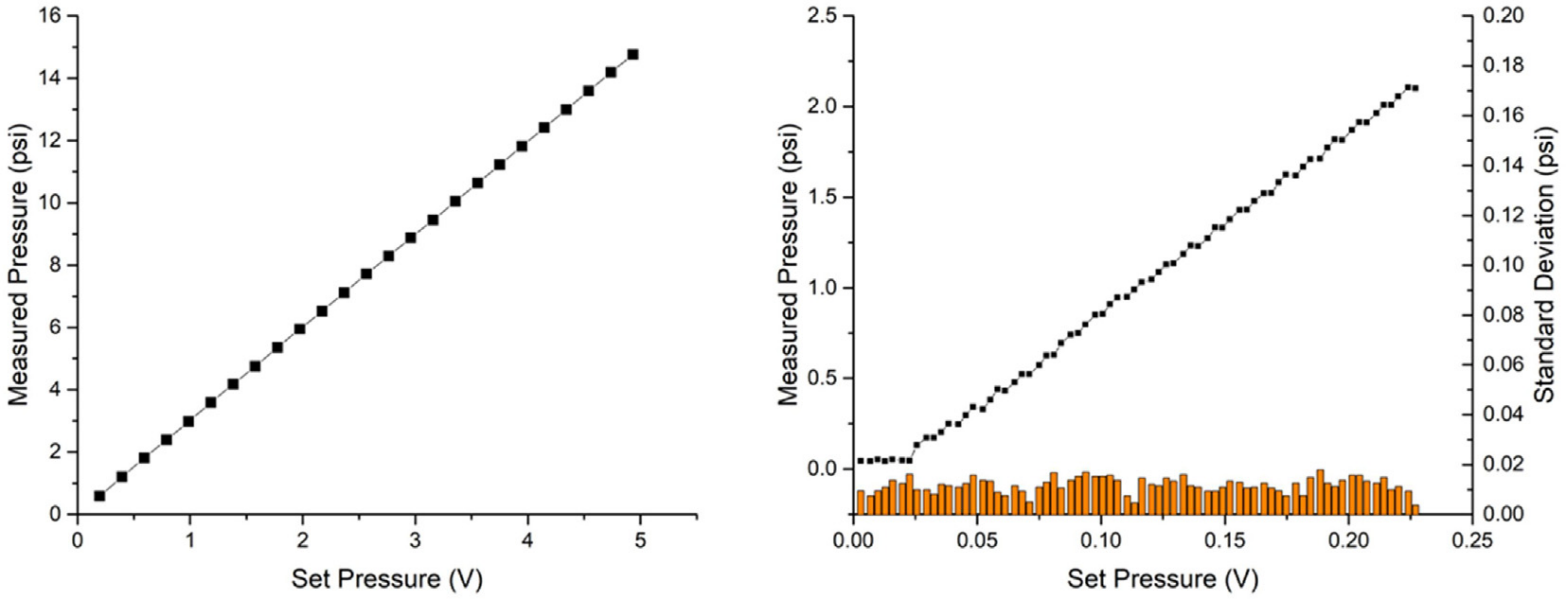
Left: Set pressure (in V) versus measured pressure (in psi) for a 15-psi Model 415 controller. Right: low range measurement with a 50-psi model 415 controller.

To demonstrate the board's ability to precisely control and measure pressure, a Parker Model 415 electronic pressure regulator was connected to J2. The resulting output pressure was measured with one of the two onboard pressure sensors. Two Model 415 were tested, one with a full-scale range of 15 psi and another with 50 psi. Fig. 17, left, depicts the measured linear dependency of the set versus measured pressure over the full range of the 15-psi controller. On the right, the measurement has been repeated for the 50-psi controller while focusing on the very end (<2.5 psi) of the controlled pressure range. Even at these low pressures, there is still a linear dependency of the control voltage versus pressure, with an average standard deviation of < 0.02 psi. Again, depending on the pressure controller and sensor used, the firmware needs to be adjusted to make sure the pressure values are scaled correctly. The firmware controls and reads both the pressure sensor and pressure controller in bit integers. If the user prefers different units, such as kPa or mBar, this can also directly be implemented in the scaling factor.

### High Voltage Power Supply Interface

**Fig. 18:**
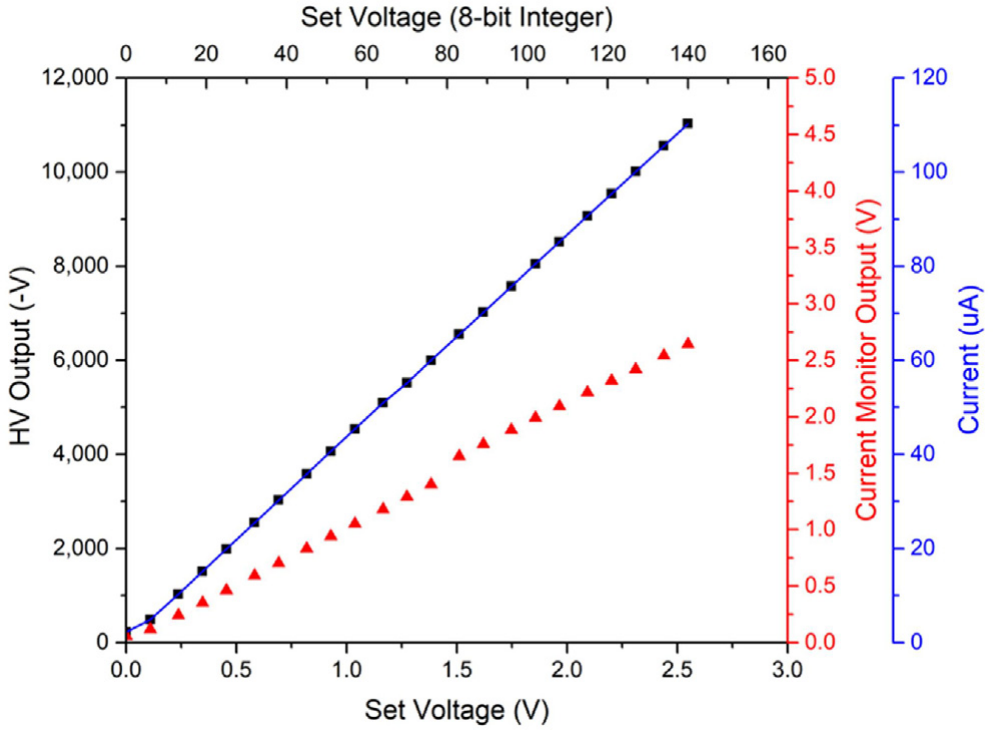
Set voltage (both in V and bit) vs. measured HV output (in V, black squares, left axis), and versus measured current monitor output (in V, red triangles, right axis). The current monitor output voltage can be calibrated using a resistor and Ohms-Law to express the current in uA (blue line, far-right axis).

A high voltage power supply can be connected to J_HV and controlled through the provided firmware. For the experiments below, a UM20N4/C/T/M HVPS from Spellman was used. Once enabled, the output voltage of the HVPS can be controlled via the interface's HV_VSET pin. As depicted in Fig. 18, the output voltage linearly depends on the set voltage. The high voltage was measured used a Fluke 174 multimeter equipped with a high voltage probe (Fluke Corporation, WA, USA). To measure and calibrate the current output of the HVPS, a 100 MΩ resistor was installed between the HV output and ground, and the current monitor output was measured versus various voltages. In combination with the measured output voltage, the known resistor value, and Ohms-Law, the resulting current can be calculated based on the current monitor output and expressed in uA. As mentioned above, if an HVPS with a different voltage and current range is used, this calibration needs to be repeated, and the firmware adjusted to represent the output correctly in HVPS.cpp lines 39 and 49 for current and voltage, respectively.

## Declaration of Competing Interest

The authors declare that they have no known competing financial interests or personal relationships that could have appeared to influence the work reported in this paper.

## Footnotes

1 https://www.arduino.cc/

2 https://www.putty.org/

3 https://www.emtec.com/zoc/

4 https://www.python.org/

5 https://www.ni.com/en-us/shop/labview.html

6 https://www.mathworks.com/products/matlab.html

7 https://platformio.org/

8 https://www.pjrc.com/teensy/td_pulse.html

## Footnotes

9 https://www.arduino.cc/

10 https://www.pjrc.com/teensy/td_download.html

11 https://osf.io/qhsb3/

## References

[1] Whitesides G.M. (2006). The origins and the future of microfluidics. Nature.

[2] Dittrich P.S., Manz A. (2006). Lab-on-a-chip: microfluidics in drug discovery. Nature reviews. Drug Discovery.

[3] da Costa E.T. (2014). Getting started with open-hardware: Development and control of microfluidic devices. Electrophoresis.

[4] White J.A., Streets A.M. (2018). Controller for microfluidic large-scale integration. HardwareX.

[5] Watson C., Senyo S. (2019). All-in-one automated microfluidics control system. HardwareX.

[6] Brower K., Puccinelli R.R., Markin C.J., Shimko T.C., Longwell S.A., Cruz B., Gomez-Sjoberg R., Fordyce P.M. (2018). An open-source, programmable pneumatic setup for operation and automated control of single-and multi-layer microfluidic devices. HardwareX.

[7] Unger M.A. (2000). Monolithic microfabricated valves and pumps by multilayer soft lithography. Science.

[8] Mora M.F., Kehl F., Tavares da Costa E., Bramall N., Willis P.A. (2020). Fully Automated Microchip Electrophoresis Analyzer for Potential Life Detection Missions. Anal. Chem..

[9] Kehl, F., et al, Open-Source Lab Hardware: Driver and Temperature Controller for High Compliance Voltage, Fiber-coupled Butterfly Lasers, under review in HardwareX. 10.1016/j.ohx.2021.e00240 PMC9123447 35607676

[10] Cretu, V.F., Kehl, F., et al, Open-Source Lab Hardware: Low Noise Adjustable Two-Stage Gain Transimpedance Amplifier with DC Offset, under review in HardwareX. 10.1016/j.ohx.2021.e00233 PMC9123464 35607697

[11] Weinberger, R., Practical capillary electrophoresis. 2000: Elsevier.

[12] Fu L.-M., Yang R.-J., Lee G.-B., Liu H.-H. (2002). Electrokinetic injection techniques in microfluidic chips. Analytical chemistry.

[13] Everaerts, F.M., J.L. Beckers, and T.P. Verheggen, Isotachophoresis: theory, instrumentation and applications. 2011: Elsevier.

[14] Pollack M.G., Shenderov A.D., Fair R.B. (2002). Electrowetting-based actuation of droplets for integrated microfluidics. Lab on a Chip.

